# A giant ovarian mucinous tumor in a 58-year-old postmenopausal patient with persistent abdominal pain and high serum levels of CA 19-9

**DOI:** 10.11604/pamj.2020.37.76.25932

**Published:** 2020-09-18

**Authors:** Francesk Mulita, Nikoleta Oikonomou, Levan Tchabashvili, Elias Liolis, Ioannis Kehagias

**Affiliations:** 1 Department of General Surgery, General University Hospital of Patras, Patras, Greece,; 2 Department of Pediatrics, Neonatal Intensive Care Unit, General University Hospital of Patras, Patras, Greece,; 3 Department of Internal Medicine, Division of Oncology, General University Hospital of Patras, Patras, Greece

**Keywords:** Tumor, ovarian, mucinous, CA 19-9

## Abstract

Ovarian cancer is the seventh most commonly diagnosed cancer among women in the world and epithelial ovarian cancer is the most predominant pathologic subtype. Tumor markers are widely used in clinical practice to determine therapeutic efficacy, to detect recurrence and to predict prognosis in known cancers. CA-19-9 antigen is mainly elevated in cases of gastrointestinal tract malignancy, including of the pancreas, colorectum, and biliary tract. However, CA 19-9 antigen can also be elevated in ovarian mucinous neoplasms. We report herein the case of a 58-year-old woman who presented with an abnormally high level of CA 19-9 antigen associated with ovarian mucinous borderline tumor.

## Introduction

Ovarian cancer is the sixth most common tumor in women and constitutes 4% of all cancers diagnosed in women [1]. Epithelial ovarian cancer is the most common type and occurs in about 90% of cases. The rest 10% originate from germ cells, from sex cords, and from ovarian stroma cells. More than 75% of epithelial ovarian cases are of the serous histological type, while mucinous, endometrioid, clear cell, Brenner, and undifferentiated lineage cancers are less common. Difficulties in early detection contribute to the high mortality rate. Most patients (> 75%) are diagnosed at a more advanced stage, with a 5-year survival rate less than 30% [2]. Biochemical and radiological assistance should always be implemented for earlier diagnosis and thereby to reduce the burden of morbidity and mortality. Understanding the origin of ovarian cancer and the specific histological types is of prime importance in diagnosing as well as offering the specific treatment [3]. Tumor markers are widely used in clinical practice to determine therapeutic efficacy, to detect recurrence and to predict prognosis in known cancers. Markers for ovarian cancer include CA-125 antigen, CA-15-3 antigen, and carcinoembryonic antigen (CEA) [4]. CA-19-9 antigen is a monosialoganglioside secreted by mucinous tumors of the gastrointestinal tract, including those of the pancreas and biliary tree. CA-19-9 antigen can be elevated in many malignancies, including colorectal carcinoma, pancreatic adenocarcinoma and epithelial ovarian carcinoma [5, 6]. Herein we report a case of an abnormally high CA-19-9 level associated with a giant ovarian tumor.

## Patient and observation

A 58-year-old postmenopausal woman presented to our Emergency Department with complaints of massive abdominal distention which started gradually 8 months ago. The patient also complained of difficulty in breathing and ambulation. There were no other gastrointestinal, urinary, or gynecological symptoms. The woman had a significant past medical history of hyper-tension and her past surgical history included one cesarean section 10 years ago. Her family history is negative for malignant ovarian and breast cancer in first-degree relatives.

**Figure 1:**
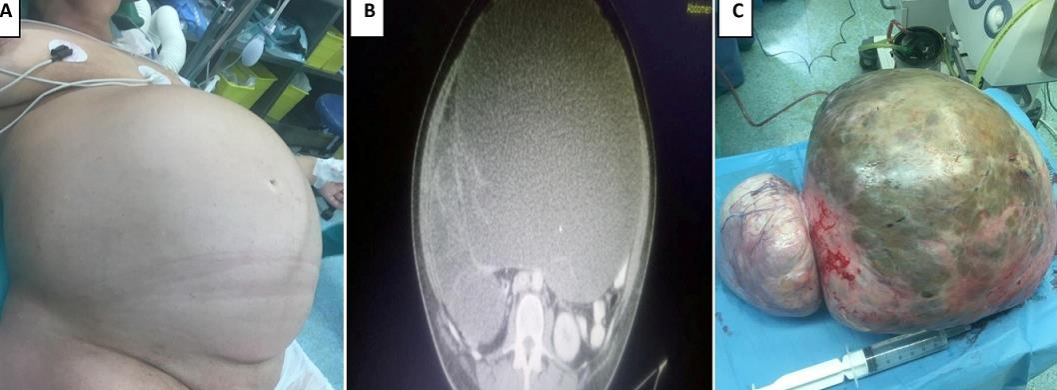
A) giant abdominal mass; B) abdominal and pelvis CT revealed a giant mass arising from the right adnexa; C) completely resection of the ovarian mass that weighed 15,4kg and measured 44x39x19cm

On physical examination, the patient´s abdomen was grossly distended, hard and tense on palpation (Figure 1A). Blood analysis revealed hemoglobin of 12.6 g/dL, 6330 leukocytes and 351000 platelets. All tumor markers were within normal limits. However CA 19-9 level was more than 953 U/mL (normal range: 0-37 U/mL). An abdominal and pelvis scan (CT) with contrast was done, which revealed a huge pelvi-abdominal mass measuring more than 45 x 35cm (Figure 1B).

The patient was operated for exploratory laparotomy with removal of right ovarian mass with total abdominal hysterectomy with bilateral salpingo-oophorectomy with complete omentectomy and appendectomy (Figure 1C). All other abdominal and pelvic organs were normal and para-aortic lymph nodes were not palpable. The 44cm x 39cm x 19 cm lobulated tumour weighing 15.4 kg had a thick wall with solid and cystic areas filled with mucinous fluid. Her final histopathology report was suggestive of borderline mucinous tumor of right ovary and cystadenoma of left ovary. The FIGO stage was pT1c2. The patient had no intra-operative or postoperative complications. On day 6 of surgery, patient was discharged and referred to oncology department for further management. She received adjuvant chemotherapy and was discharged home in healthy condition. Four months following the surgery, the CA 19-9 levels had regressed to normal range. The patient has been symptom-free thereafter and the CA 19-9 levels have also remained normal until date, i.e. ten months past the surgery.

## Discussion

Women with abdominal-pelvic masses constitute a challenging condition in general practice because the clinical features and findings from physical examination are usually nonspecific. The clinical application of tumor markers, such as serum concentration of CA 125, AFP and hCG is of great help not only as diagnostic aid but also in monitoring efficacy of any treatment modality like chemotherapy, radiotherapy or surgical resection [4, 7]. Carbohydrate antigen 19-9 (CA 19-9) (a sialylated lewis glycoprotein antigen), a commonly used marker of benign and malignant hepatobiliary and pancreatic conditions, has also been found to be raised in some ovarian neoplasms [8]. The diagnosis of epithelial ovarian cancer is difficult because the symptoms and signs are vague and nonspecific. A combination of transvaginal ultrasonography and serum CA-125 antigen level can contribute to early diagnosis of epithelial ovarian cancer [4, 6, 9]. In primary ovarian mucinous tumors, CA-19-9 antigen is used as a marker because CA-125 antigen is not elevated in most primary ovarian mucinous neoplasms [10]. Tumor markers including CA-19-9 and CA-125 antigen are widely used for prediction of the characteristics of ovarian masses. Abnormal levels of these markers can lead to unnecessary medical intervention and patient anxiety. Therefore, clinical information and antigen testing results should be interpreted carefully [4, 6].

## Conclusion

Ovarian cancer will continue to be a risk for woman, as the majority of patients will not be diagnosed at an early stage. Physicians must maintain heightened awareness and index of suspicion when approaching an ovarian mass, because the early diagnosis can improve the patient´s prognosis.
